# The Secretary's Rôle at a Board Meeting

**Published:** 1911-12-09

**Authors:** 


### THE SECRETARY'S RÔLE AT A BOARD MEETING.

By AN INSTITUTIONAL SECRETARY.

As one who has had the opportunity of attending a number of hospital board meetings at different institutions, it is clear that the subject of the secretary's rôle when a board meeting is in progress is one of very debatable interest.

To begin with, the secretary should, of course, always await instructions from the chairman before beginning to read the minutes. Immediately he has finished reading he should wait a second or two to hear if any clauses in the minutes are not in accordance with what the members present agree upon as accurate. He should then hand the book to the chairman for signature. It has been said by one or two secretaries that it is their custom to stand when reading the minutes at a board meeting. This practice is one to be followed, for it ensures that the reader will be heard. It is usual for the secretary to stand when reading or speaking at all properly regulated Boards and Committees. Speakers at meetings should always stand up.

The reading of the minutes cannot be too carefully done. Some secretaries say: "Oh, anybody can read the minutes." But, reading so that all who hear must comprehend and follow, and reading aloud in a haphazard and unintelligent manner, are two wholly different if common enough performances.

### Talkative Secretaries.

Some secretaries, it seems to me, make the mistake of having far too much to say at a board meeting. A secretary should answer, as far as he can, and as concisely as he can, every question that is put to him by the chairman, or by any other member of the board. He should, naturally, have prepared himself for any question on any of the subjects which are down on the agenda. He will also save a good deal of time and discussion if he prepares beforehand any letters which he thinks it may be thought necessary for the board to send on any point arising out of the day's business. It does not matter much whether the letters are approved by the board or whether they are not; they can be amended. It is always easier for a board to draft a letter when they have had before them a specimen of what might be sent.

There are secretaries who have actually attempted to "bully" their boards. They have done this, presumably, because they have outgrown their position, or because they have been foolish enough to suppose that they are indispensable. It does not pay to act the part of "bully" to a board, even if the board is weak enough to tolerate such behaviour. Hospital secretaries, when they know their business thoroughly, frequently have very kind, or even very flattering, remarks addressed to them by members of their boards, but such observations should always be taken with humility (and with a grain of salt), for no hospital secretary should forget that honorary officials are desirous, excusably, of keeping on good terms with salaried officials, inasmuch as the latter are likely to be approached by strangers of all classes respecting the actual work done by the honorary members.

At every board meeting, if it is attended by any number between six and eighteen, there are sure to be one or two who are inattentive and who cough or fidget while business is being transacted. I have a little way of getting over this drawback: I never fail to put upon the board-room table a copy of *Punch* and other publications, which are likely to attract the eyes of those who will not concentrate their minds on the subjects under discussion. This is a perfectly harmless way of meeting a forgivable human weakness. I think every secretary will agree with me that when he takes pains to read clearly and lucidly he is only doing bare justice to himself when he endeavours to abolish fidgeting.

The secretary should invariably take his cue from the chairman. If a chairman and another member of the board ask questions at the same moment, it is the secretary's clear and obvious duty to reply to the chairman, whether he gets an opportunity later on or not of answering the lesser member of the board. The other member has his remedy, if he repeats his question to the chairman, or to the secretary. A secretary should never be forgiven for wasting time at a meeting. He should act as if under an obligation to every member of his board for their attendance. On the other hand, a secretary should not rush business through, without adequate and fitting deliberation on the part of those who are, after all, responsible. It is all very well for a secretary to imagine that he can deal individually with the matter as expediently as his board, but he should not forget that if anything goes wrong the trouble will assuredly recoil upon himself.

### The Secretary's Part in a "Scene."

In the unhappy event of a "scene" occurring at a board meeting, the secretary is wise if he "sits tight" and keeps his eyes on his blotting-paper. He must not even appear to have a leaning one way or the other, for long after the scene is over and perhaps forgotten, it is likely that at least one member present saw what happened and made a mental note of the official's favouritism.

Boards soon get to know the habits of their secretaries, and, therefore, it is well if all bad traits are avoided. The secretary should take equal board-room trouble over Mr. Brown and Mr. Smith. A secretary should try and let it be seen that he has a high opinion of his board's judgment, though if in his heart he believes his own judgment on a certain point to be sounder, he should not be afraid of saying so, in a delicate and suggestive way. If a secretary's personal view is ventilated guardedly and tactfully, even if it is an opposite view to that of the majority, the secretary will, at any rate, gain a reputation for not being too idle to think, but a too cocksure manner is always irritating, more especially if it is exhibited before one's masters.

